# Growth of Well-Aligned InN Nanorods on Amorphous Glass Substrates

**DOI:** 10.1186/s11671-016-1482-x

**Published:** 2016-05-26

**Authors:** Huijie Li, Guijuan Zhao, Hongyuan Wei, Lianshan Wang, Zhen Chen, Shaoyan Yang

**Affiliations:** Key Laboratory of Semiconductor Materials Science, Beijing Key Laboratory of Low Dimensional Semiconductor Materials and Devices, Institute of Semiconductors, Chinese Academy of Sciences, P. O. Box 912, Beijing, 100083 People’s Republic of China; University of Chinese Academy of Sciences, Beijing, 100049 People’s Republic of China; LatticePower (Jiangxi) Corporation, No. 699 North Aixihu Road; National High-Tech Industrial Development Zone, Nanchang, 330029 Jiangxi Peoples Republic of China

## Abstract

The growth of well-aligned nanorods on amorphous substrates can pave the way to fabricate large-scale and low-cost devices. In this work, we successfully prepared vertically well-aligned *c*-axis InN nanorods on amorphous glass substrate by metal-organic chemical vapor deposition. The products formed directly on bare glass are randomly oriented without preferential growth direction. By inserting a GaN/Ti interlayer, the nanowire alignment can be greatly improved as indicated by scanning electron microscopy and X-ray diffraction.

## Background

Besides the great successful applications in commercial light-emitting diodes and high-electron-mobility transistors [1–13], III-nitrides have also emerged as promising candidates for applications in photovoltaics (PVs) [14–16], water splitting [17–19], and piezotronics [20] because of their remarkable properties including wide bandgap range (0.68~6.2 eV) [21–23], high chemical inertness [24], and large piezoelectric coefficients [25]. Traditionally, III-nitrides are formed on single-crystalline substrates (sapphire, Si, SiC, etc.) which have a good epitaxial relationship with them. However, these substrates are expensive and have small wafer size, limiting their usage in larger scale fabrication (greater than single-crystal substrates). In future possible applications of III-nitrides such as flat panel display, PVs, and hydrogen production, large-size and cheap substrates are the key factors to reduce the system cost. In this regard, growth of III-nitrides on glass or common metal substrates is highly desired because these substrates have large wafer size and low manufacturing cost. Unfortunately, due to the lack of global epitaxy, it is very difficult to obtain single-crystalline semiconductor films on these substrates.

Nanostructures, on the other hand, are much easier to grow on glass or metal substrates because of their small lateral size. It has been reported that nanowires of various materials have been successfully obtained on such substrates [26–30]. However, these nanowires were usually randomly distributed without any preferential orientation, hindering the subsequent heterostructure formation and device processing. Wölz et al. [31] found that vertically aligned GaN nanowires can be grown in a self-induced way on a sputtered Ti film by plasma-assisted molecular beam epitaxy (PAMBE). Zhao et al. [32] reported the growth of vertically aligned GaN nanowires on SiO_x_ buffer layers by MBE. But the Ti or SiO_x_ layers were actually deposited on single-crystalline sapphire or Si substrates, it cannot lead to a conclusion that vertically aligned GaN nanowires can be spontaneously formed on bulk metal or glass substrates. Moreover, the present works mainly focused on the growth of GaN nanostructures, other III-nitride materials (InN, AlN) are much less concerned although they have similar importance. For example, InN nanorods have been demonstrated to be ideal templates for the growth of GaN and AlN nanostructures [33, 34]. By depositing GaN (AlN) epilayers on the InN nanorods and thermally remove the InN nanorods, GaN (AlN) nanotubes can be easily formed. Unlike the most used ZnO nanorod templates for GaN (AlN) nanotubes, InN can be grown in the same furnace with those materials without being exposed to the air, which can simplify the growth process and avoid the template contamination. Therefore, if well-aligned InN nanorods can be grown on amorphous substrates, it is facile to obtain well-aligned GaN and AlN nanostructures on these substrates.

In this work, we demonstrated that vertically well-aligned InN nanorods can be grown on amorphous glass substrate by metal-organic chemical vapor deposition (MOCVD) using a GaN/Ti interlayer. The nanorods were formed by introducing Zn dopant in the growing process. Direct growth of InN on the glass substrate without any interlayer resulted in the random aligned nanorods without any preferential orientation. By introducing a thin-film Ti and GaN as the pre-orienting and nucleation layers, we can obtain well-aligned InN nanorod arrays along the *c*-axis crystal orientation.

## Methods

Three different types of templates (A, B, and C) were prepared on fused-silica glass wafers. Templates A, B, and C had Ti, LT-GaN/Ti, and HT-GaN/LT-GaN/Ti layers, respectively. The Ti pre-orienting layer (100 nm) was electron-beam evaporated at room temperature. The LT-GaN nucleation layer (200 nm) was grown at 600 °C by a homemade MOCVD system, which has been described by previous researchers in our group [35]. HT-GaN layer was grown at 1050 °C on the LT-GaN/Ti layer. For the growth of GaN, trimethylgallium (TMGa) and NH_3_ were used as the Ga and N source, respectively. H_2_ was used as the carrier gas. The growth pressure for GaN was 50 Torr.

InN nanorods were grown under atmospheric pressure at 520 °C using trimethylindium (TMIn) and NH_3_ as the precursors. Diethylzinc (DEZn) was introduced as the dopant in the InN nanorod growth process. N_2_ was used as the carrier gas. The flow rates of TMIn and ammonia are 14 μmol/min and 3 SLM, respectively. The growth time for InN nanorods was 40 min. After the growth, the TMIn flow was cut off and the furnace was cooled down to the room temperature. NH_3_ was maintained during cooling down in order to prevent the decomposition of InN.

The morphologies of the samples were examined by scanning electron microscopy (SEM; Nova NanoSEM 650). The crystal structure of the products was characterized by X-ray diffraction (XRD; Philips X’pert Pro X-ray diffractometer) with Cu Kα radiation of 0.15406 nm. The nanorods grown on template C were dispersed onto copper grids possessing an amorphous carbon film and further characterized with a high-resolution transmission electron microscope (HR-TEM; FEI TECNAI F30, 300 kV).

## Results and Discussion

**Fig. 1:**
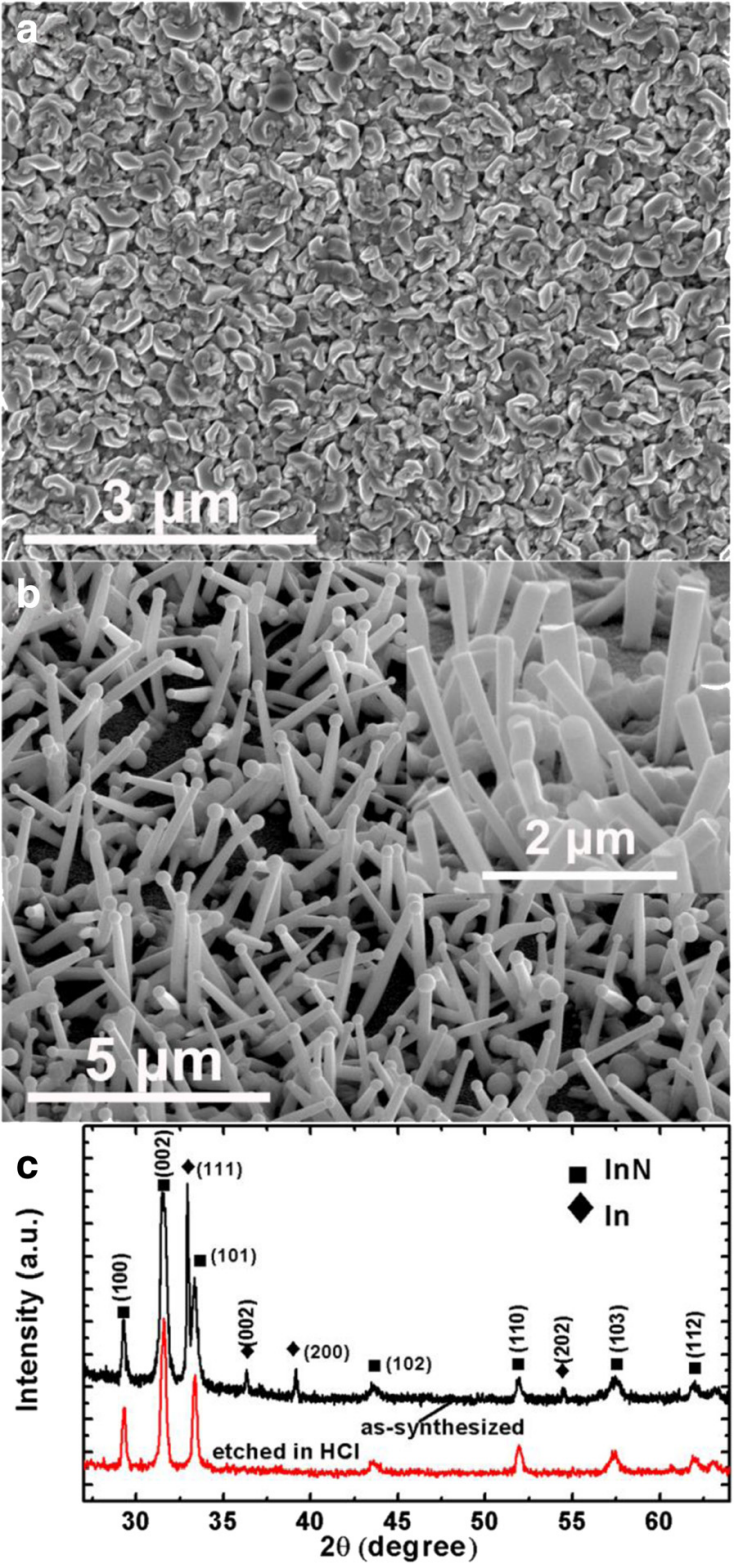
**a**, **b** The InN grown on bare glass substrates without and with Zn doping, respectively. **c** XRD spectra of the as-synthesized and HCl-etched InN nanorods

Figure 1a shows the SEM image of the InN grown on glass substrates without Zn doping. The product exhibits non-continuous film-like morphology, no nanorod is observed. By introducing Zn source in the growth of InN, we can obtain dense InN nanorods on bare glass substrates, as shown in Fig. 1b. Each nanorod has a droplet on the end, which can be etched away in diluted HCl solution (Fig. 1b inset). In the MOCVD growth of InN nanorods on single-crystalline sapphire substrates, Zn dopant was found to be crucial to the formation of rod-like structure by limiting the lateral growth of InN [36, 37]. The nanorod density and diameter were found to be influenced by the DEZn source flow rates. In this work, the DEZn flow rate was kept at an experience value of 0.7 μmol/min. More detailed works about the influence of the Zn concentration on the products will be carried in the future. Nevertheless, we demonstrated that InN nanorods can also be grown on amorphous glass through MOCVD with the assistance of Zn dopant. However, the nanorods grown on bare glass substrates do not have a preferential growth orientation due to the lack of a global epitaxial relationship between InN and the substrate. The XRD spectra of the InN nanorods are shown in Fig. 1c. The spectrum of the as-grown nanorods consists of various diffraction peaks from the wurtzite-type InN (Joint Committee of Powder Diffraction Standards (JCPDS) card 79-2498) and the metal indium (JCPDS: 85-1409). After being dipped in HCl solution, the diffraction peaks correspond to the metal indium disappeared, which indicates the droplets on the InN nanorods are metal indium droplets.

**Fig. 2:**
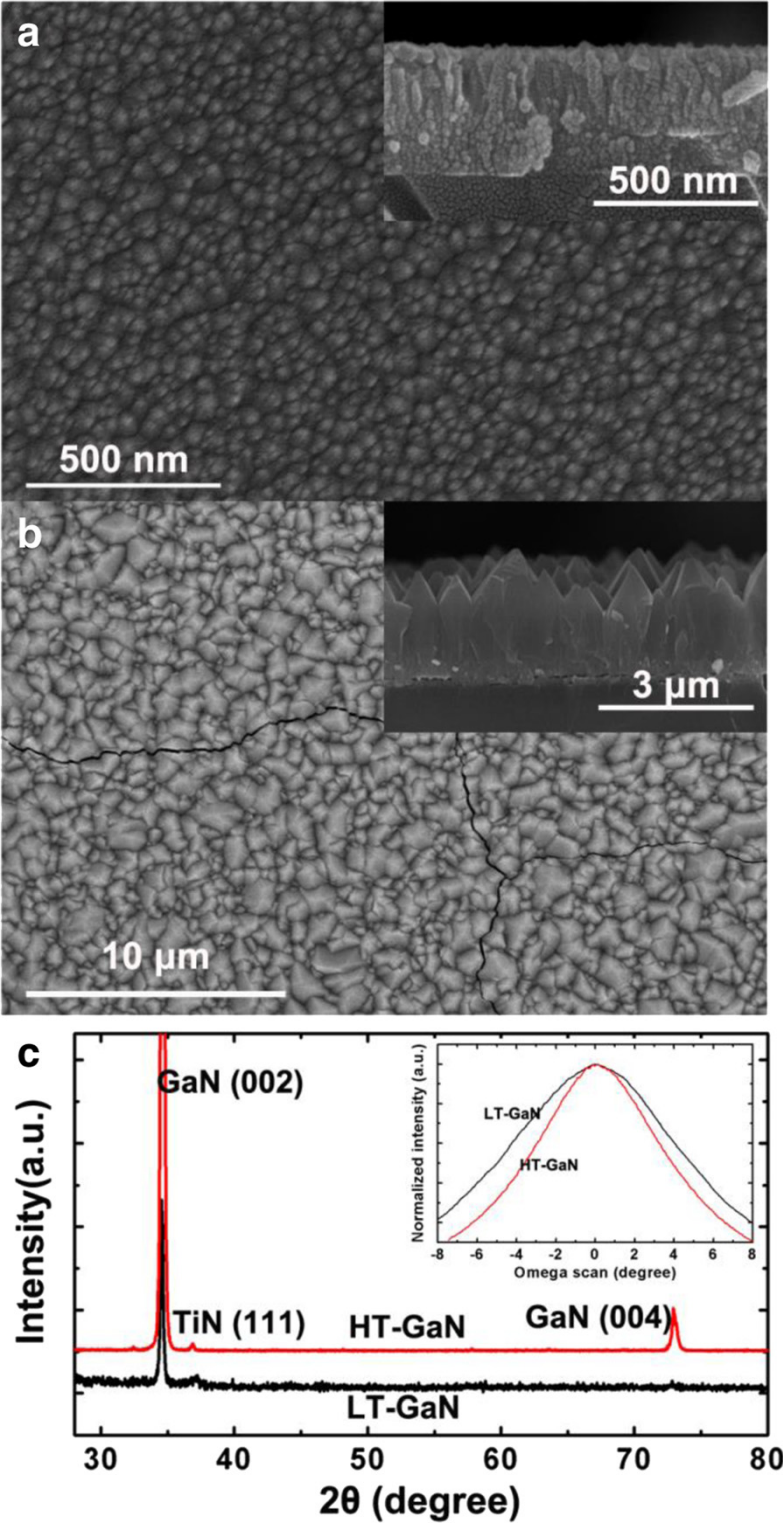
**a**, **b** SEM images of the LT- and HT-GaN layers grown on Ti/glass. *Insets* in **a**, **b** are the cross sections of the products. **c** XRD spectra of the LT- and HT-GaN layers. *Inset* is the GaN (002) rocking curves of the two samples

To improve the nanorod alignment, we prepared three different templates, namely Ti (A), LT-GaN/Ti (B), and HT-GaN/LT-GaN/Ti (C) layers. The Ti film (100 nm) was electron-beam evaporated at room temperature. The LT-GaN layer (200 nm) was grown at 600 °C by MOCVD. HT-GaN layer was grown at 1050 °C on the LT-GaN/Ti layer. For the growth of GaN, trimethylgallium (TMGa) and NH_3_ were used as the Ga and N source, respectively. H_2_ was used as the carrier gas. The chamber pressure for GaN growth was 50 Torr. The deposited Ti film was composed of columnar grains which are orientated along the *c*-axis as characterized by XRD (not shown). The preferential crystalline orientation was often observed in the evaporated or sputter-deposited Ti films, which was due to that the (001) facet of Ti has the lowest surface energy [38–41]. In previous works [31, 42], it was found that the Ti layer would be turned into cubic TiN in the presence of N source. The resulted TiN layer exhibited a [111] out-of-plane orientation and a $\left\langle 1\overline{1}0\right\rangle$ in-plane orientation. And it was found that wurtzite GaN and cubic TiN has an epitaxial relationship: $\mathrm{G}\mathrm{a}\mathrm{N}(001)\left\langle 110\right\rangle \left\Vert \mathrm{TiN}\right.(111)\kern0.24em \left\langle 1\overline{1}0\right\rangle$. Therefore, the GaN layer grown on the (111) TiN film would have a *c*-axis orientation. The SEM images of templates B and C are shown in Fig. 2a, b, respectively. We can see that the LT-GaN grown on Ti film has a columnar morphology with a typically lateral size of about 50 nm, which is similar to the results obtained by Choi et al. [38] The XRD pattern of template B shows only two diffraction peaks correspond to the (002) plane (34.56°) of wurtzite GaN and (111) plane (36.8°) of cubic TiN, as shown in Fig. 2c. The appearance of the TiN (111) peak was due to the nitridation of the Ti film by NH_3_. Since no other diffraction peak corresponds to GaN was observed, it can be inferred that the LT-GaN grown on Ti film is oriented along the *c*-axis.

After growth of HT-GaN on the LT-GaN/Ti layers, the columnar grains evolved to larger aligned pyramids which have lateral sizes from several hundred nanometres to several micrometers (Fig. 2b). It seems that the pyramids cannot coalesce into a single-crystal film even after long time growth (3 h), perhaps due to the lack of a same in-plane crystallographic arrangement between the pyramids. Cracks were generated in the HT-GaN layers because the fused glass has much smaller thermal expansion coefficient than GaN [43, 44]. The XRD pattern of template C is similar to that of template B but has much stronger intensity, as shown in Fig. 2c. XRD rocking curves of templates B and C are shown in Fig. 2c inset, we can see that the (002) preferred orientation of HT-GaN is improved as compared with the LT-GaN.

**Fig. 3:**
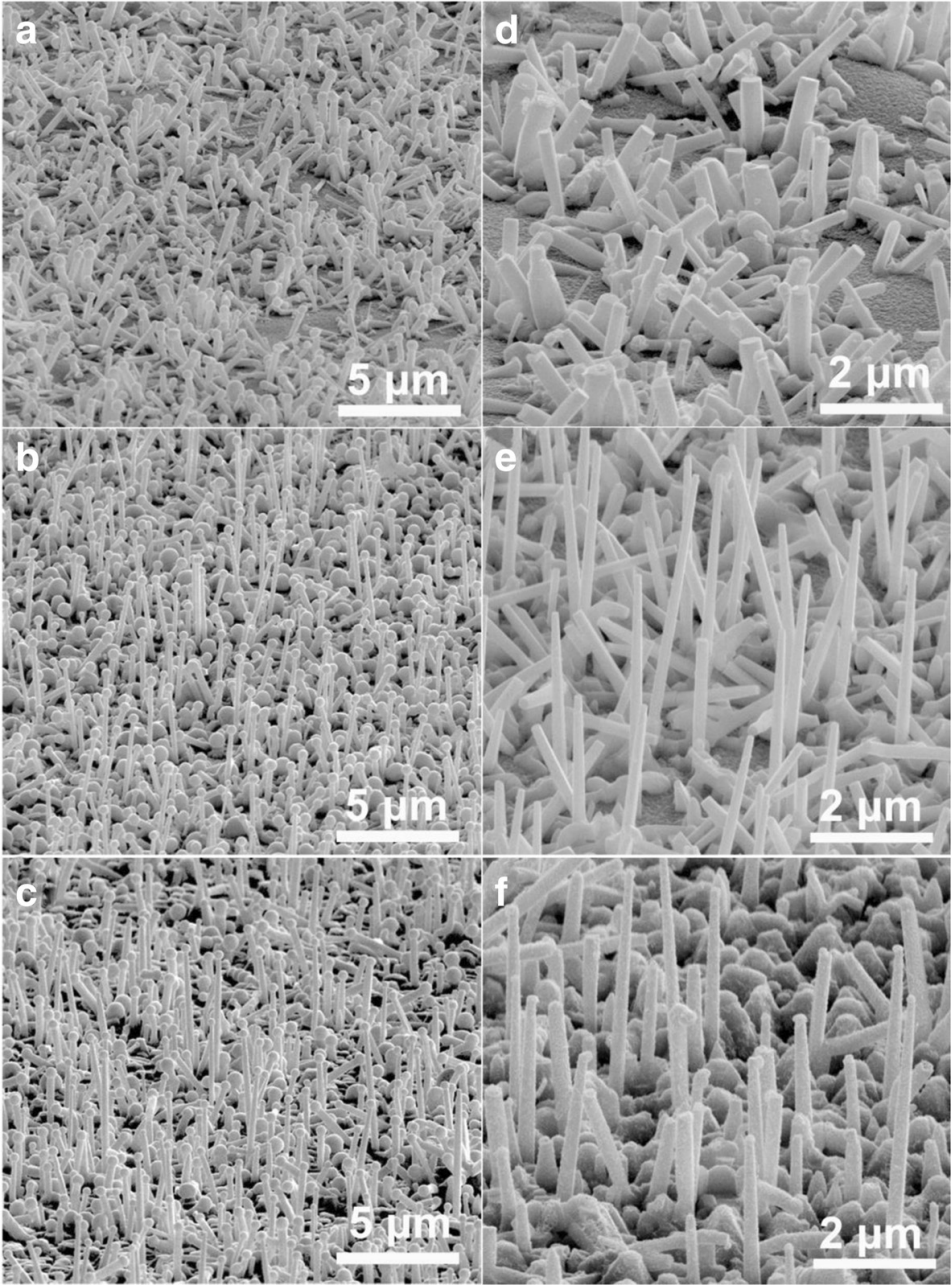
**a**–**c** The InN nanorods grown on templates A–C, respectively. **d**–**f** Enlarged view of the HCl-etched nanorods in **a**–**c**, respectively

**Fig. 4:**
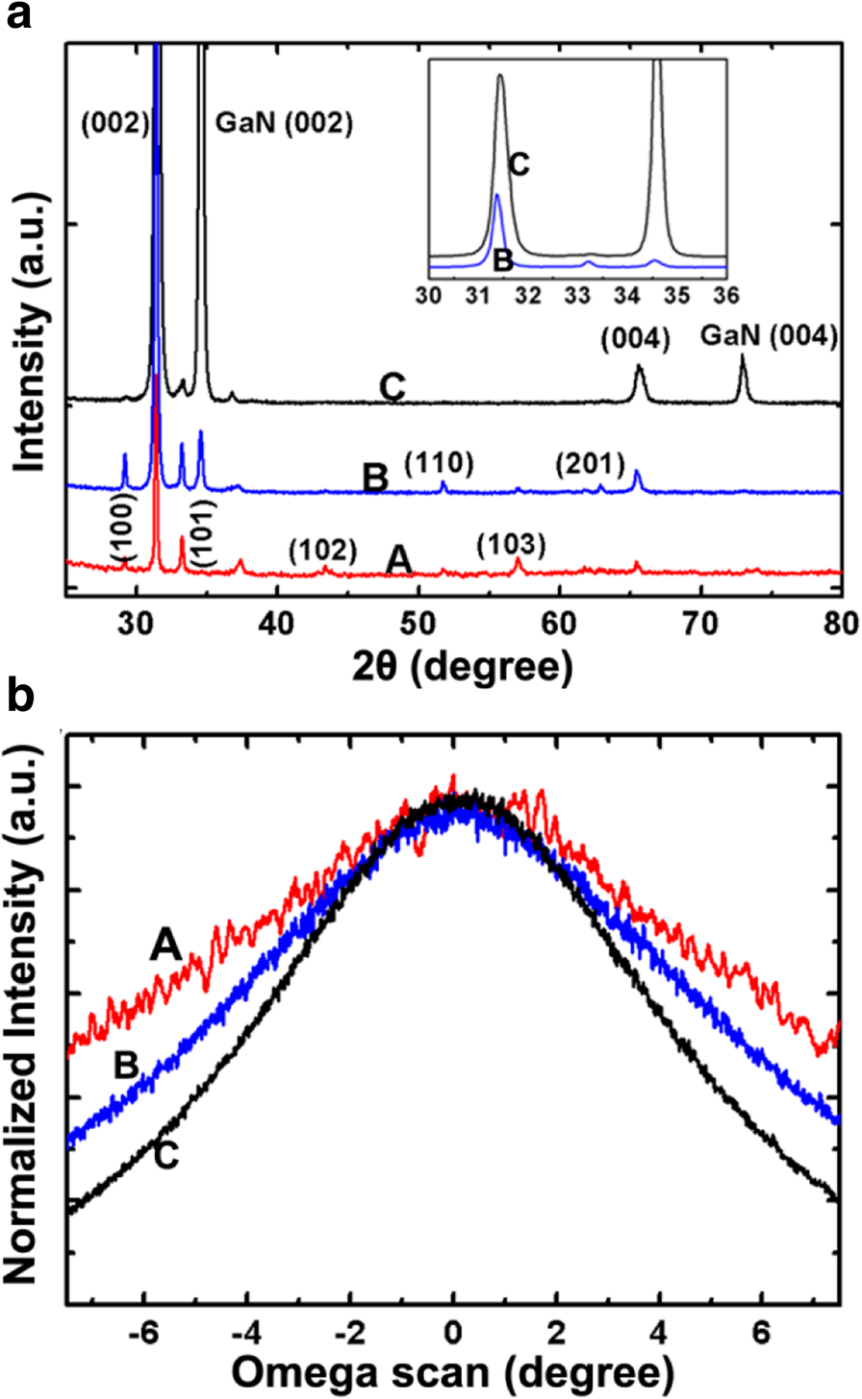
**a** XRD spectra of the HCl-etched InN nanorods grown on templates A–C. *Inset* is the enlarged view of the spectra of B and C from 30° to 36°. **b** (002) InN rocking curves of the nanorods grown on templates A–C

The InN nanorods grown on templates A–C are shown in Fig. 3. Different from the GaN epilayers, the InN nanorods grown on the Ti films did not show obvious better alignment than those grown on the bare glass substrates (Fig. 3a, d). Various diffraction peaks corresponding to different InN planes are still observed. Thus, we can conclude that a single Ti layer is not able to improve the alignment of InN nanorods obviously. From Fig. 3b, e, we can see that the InN nanorods grown on template B have much better alignment than those grown on bare glass or template A. Most of the nanorods are vertically aligned with the *c*-axis growth orientation, as indicated by the XRD spectrum (Fig. 4a). However, other diffraction planes of InN, such as (101), (110), and (201) are still observed although the intensity of such peaks are much weaker than the (002) plane. The reason might be due that the growth orientation of the LT-GaN columns is not ideally align along *c*-axis, and the alignment of the subsequently grown InN nanorods is further worsen.

The nanorods grown on the HT-GaN/Ti interlayer have a good orientation alignment, as indicated by Fig. 3c, f. Besides a few of tilted InN nanorods, the products are aligned vertically to the substrate. The appearance of the tilted nanorods might due to fact that the template C is composed of pyramids rather than being a flat film. By comparing Fig. 3e, f, we can see that the nanorods on template C have better alignment than those grown on template B. XRD spectrum indicates that the nanorods are grown along the *c*-axis of wurtzite InN. Although a small peak corresponds to (101) InN is observed, it is very weak as compared to the (002) InN peak (Fig. 4a inset). The (002) XRD rocking curves of the InN nanorods grown on templates A–C are shown in Fig. 4b, which also prove that the products grown on template C have the best preferential orientation. There was a previous report on the growth of InN nanorods on glass substrates by MBE using an AlN interlayer. The products are well aligned with a preferential [002] growth direction. However, nanorods with other growth directions might exist as revealed by the various XRD peaks [45]. In this work, we proved that a HT-GaN/Ti interlayer is more proper to obtain well-aligned nanorods with the same crystal orientation.

**Fig. 5:**
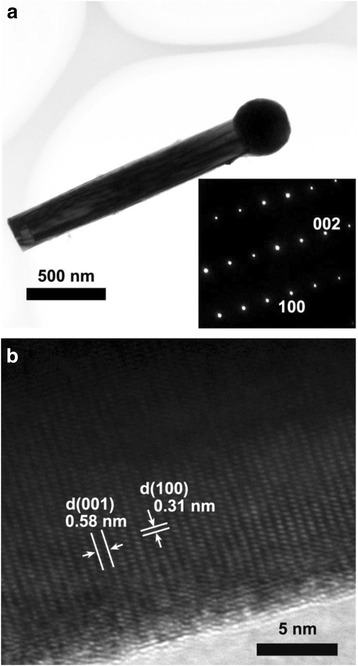
**a** TEM image of an InN nanorod grown on template C. *Inset*: SAED pattern taken along the [110] zone axis. **b** HRTEM image of the InN nanorod

Figure 5a shows the TEM images of the nanorods grown on template C. The nanorod is about 200 nm in diameter and 2 μm in length. The nanorod have smooth and abrupt side wall with an In droplet on the end. The corresponding selected area electron diffraction (SAED) pattern is shown in the inset of Fig. 5a, which was taken along the [110] zone axis of the nanorod. It shows that the InN nanorod is single crystalline. Figure 5b is the high-resolution lattice image. The interplanar distances of 0.31 and 0.58 nm match with the *d*_100_ and *d*_001_ spacing of wurtzite-type InN, respectively. These lattice parameters also indicate that the InN nanorod is grown along the *c*-axis.

## Conclusions

In summary, we have successfully prepared well-aligned *c*-axis InN nanorods on amorphous glass by MOCVD. Directly growth of InN on bare glass without any interlayer results in randomly oriented nanorods. Several templates were prepared to improve the preferential orientation of the InN nanorods. It was found that the alignment of the nanorods is highly dependent on the templates. The nanorods grown on the HT-GaN/Ti template show much better alignment than those grown on Ti or LT-GaN/Ti templates. The successful growth of well-aligned InN nanorods on amorphous substrates can pave the way to fabricate large-scale and low-cost InN-based devices. It also enables the fabrication of well-aligned GaN and AlN nanostructures on glass substrates because they can be formed using the InN nanorods as templates.
